# Selection of appropriate reference genes for RT-qPCR analysis under abiotic stress and hormone treatment in celery

**DOI:** 10.7717/peerj.7925

**Published:** 2019-10-24

**Authors:** Kai Feng, Jie-xia Liu, Guo-Ming Xing, Sheng Sun, Sen Li, Ao-Qi Duan, Feng Wang, Meng-Yao Li, Zhi-Sheng Xu, Ai-Sheng Xiong

**Affiliations:** 1 State Key Laboratory of Crop Genetics and Germplasm Enhancement, Ministry of Agriculture and Rural Affairs Key Laboratory of Biology and Germplasm Enhancement of Horticultural Crops in East China, College of Horticulture, Nanjing Agricultural University, Nanjing, China; 2 Collaborative Innovation Center for Improving Quality and Increase Profits of Protected Vegetables in Shanxi, Taigu, China

**Keywords:** Abiotic stress, Celery, Expression stability, Hormone stimuli, Reference gene, RT-qPCR

## Abstract

Celery is one of the most important vegetable crop and its yield and quality is influenced by many environmental factors. Researches on gene expression not only help to unravel the molecular regulatory mechanism but also identify the key genes in the biological response. RT-qPCR is a commonly used technology to quantify the gene expression. Selecting an appropriate reference gene is an effective approach to improve the accuracy of RT-qPCR assay. To our knowledge, the evaluation of reference genes under different treatments in celery has not been reported yet. In this study, the expression stabilities of eight candidate reference genes (*ACTIN*, *eIF-4*α**, *GAPDH*, *TBP*, *TUB-A*, *UBC*, *TUB-B*, and *EF-1*α**) under abiotic stresses (heat, cold, drought, and salt) and hormone treatments (SA, MeJA, GA, and ABA) were detected. The expression stabilities of candidate genes were compared and ranked by geNorm, NormFinder, BestKeeper, ΔCt, and RefFinder programs. The results calculated by different programs were not completely consistent. Considering the comprehensive analysis results, *ACTIN* was the most stable reference gene and *TUB-B* showed the worst expression stabilities under the selected abiotic stress and hormone treatments in celery. The reliability of reference genes was further confirmed by the normalization of *CAT1* gene under drought stress. This study presented evidences and basis to select the appropriate reference genes under different treatments in celery.

## Introduction

Celery (*Apium graveolens* L.), one plant of Apiaceae, is an important vegetable and its leaves are the mainly edible organs (Li et al., 2018). Nowadays, celery is commonly consumed for its abundant nutritional values (apigenin, vitamin C, and cellulose etc.) and low calorie contents (Dianat et al., 2015). The yield and quality of celery are influenced by many environmental factors (temperature, moisture, soil salinity, and hormone) (Golldack, Luking & Yang, 2011). During plant development, many environmental stresses disturb the physiological processes and affect the growth and development (Cattivelli et al., 2008; Kosova, Prasil & Vitamvas , 2008; Wang et al., 2012; Wang et al., 2014). Phytohormones are known to be plant growth regulators and play vital roles during plant development, such as gibberellic acid (GA), salicylic acid (SA), abscisic acid (ABA), and methyl jasmonate (MeJA) (Moons et al., 1997; Wang et al., 2015; Liu et al., 2016; Galimba et al., 2019). Under abiotic stress and hormone treatments, plants generate many responsive mechanisms to relieve environmental damages (Kosova, Vitamvas & Prasil , 2014). The molecular mechanisms including physical, physiological, and biochemical responses were associated with the expressions of certain genes (Wang, Vinocur & Altman, 2003). Researches on expressions of abiotic stress-related genes provided strategies to improve the stress resistance in molecular breeding (Brikis et al., 2018; Wang et al., 2019).

The gene expression analysis was commonly applied to understand the molecular regulatory mechanisms and identify the key genes in the current molecular biology (Bustin et al., 2005; Silva et al., 2019). Quantitative real time polymerase chain reaction (RT-qPCR) has become a recognized technology for quantifying the gene expression due to its advantages of high-throughput, high-sensitivity, high-veracity, and low-cost (Gachon, Mingam & Charrier , 2004; Bustin et al., 2005; Derveaux, Vandesompele & Hellemans , 2010; Miao et al., 2019). Nevertheless, many factors including enzymatic efficiency, RNA purity, and cDNA quality may affect the accuracy and credibility of RT-qPCR results (Vandesompele et al., 2002). Several strategies were applied to ensure the accuracy of RT-qPCR. Selection of one or more suitable internal control genes, also known as house-keeping genes, has become a frequently method to normalize the gene expression (Gutierrez et al., 2008).

The house-keeping genes have been identified in many species, including Arabidopsis (Czechowski et al., 2005), rice (Jain et al., 2006), and soybean (Libault et al., 2008). House-keeping genes, e.g., glyceraldehyde-3-phosphate dehydrogenase (*GAPDH*), ubiquitin C (*UBC*), actin (*ACTIN*), eukaryotic translation initiation factor 4*α* (*eIF*-4*α*), elongation factor-1*α* (*EF*-1*α*), TATA-box binding protein (*TBP*), and tubulin (*TUB*) were widely used as reference gene to standardize the expressions of target genes (Dheda et al., 2004; Galli et al., 2013; Li et al., 2016b). However, some studies also indicated that the expressions of certain house-keeping genes under different tissues or treatments were fluctuant (Barber et al., 2005; Borowski et al., 2014; Tian et al., 2015). The unstable reference gene would significantly influence the accuracy and reliability of target gene expression quantification. The suitable reference gene for RT-qPCR analysis of celery under different tissues and developmental stages has been identified in previous study (Li et al., 2016b). To our knowledge, the appropriate reference genes for RT-qPCR analysis of celery under abiotic stress and hormone treatment have not been reported yet. Considering the roles of gene expression in the molecular biology of celery, the comparison and selection of reference genes under various experimental treatments is necessary.

Here, eight known house-keeping genes, *ACTIN*, *eIF*-4*α*, *GAPDH*, *TBP*, *TUB-A*, *UBC*, *TUB-B*, and *EF*-1*α* were selected based on the previous studies (Tian et al., 2015; Li et al., 2016b; Wu et al., 2016). The expression stabilities of candidate reference genes under abiotic stresses (heat, cold, drought, and salt) and hormone treatments (GA, SA, ABA, and MeJA) were assessed by using geNorm (Vandesompele et al., 2002), NormFinder (Andersen, Jensen & Orntoft , 2004), BestKeeper (Pfaffl et al., 2004), ΔCt (Silver et al., 2006), and RefFinder programs (Xie et al., 2012). The gene encoding catalase in celery, *CAT1*, was selected to assess the reliability of candidate reference genes under drought treatment. The current study will provide useful information for selecting suitable reference genes to conduct RT-qPCR analysis in celery under abiotic stress and hormone stimuli.

## Materials & Methods

### Plant materials and experimental treatments

The seeds of celery cultivar ‘Jinnan Shiqin’ were germinated in a petri dish at room temperature. Celery seedlings were transferred into the plastic pots with 1:1 mixture of soil and vermiculite. Seedlings were grown in an artificial climatic chamber with the condition as previously described (Feng et al., 2018c). After 8 weeks of growth, the vigorous seedlings with consistent growth were selected for experimental treatments. As for heat and cold stresses, seedlings were placed in the light incubators with temperatures of 38 °C and 4 °C, respectively. For drought and salt stresses, seedlings were irrigated with 0.5 L of PEG 6000 (20%) and NaCl (0.2 M) solution, respectively (Tian et al., 2015). As for hormones treatments, celery leaves were sprayed with 0.5 L of GA (1.4 mM), SA (1.4 mM), ABA (0.1 mM), and MeJA (0.8 mM), respectively (Chan, 2012; Zhu et al., 2013; Li et al., 2016a). All of the treatments were performed with three biological replicates. Leaf blades were collected from untreated and treated celery plants after 2 h of treatments.

### Preparation of RNA and cDNA

Total RNA was extracted from the celery samples by using Total RNA Kit (Tiangen, Beijing, China) based on manufacturer’s protocol. The concentration and quality of total RNA was measured by using a One-Drop™ spectrophotometer. The qualified RNA (1 µg) were used to synthesize cDNA by using the Prime-Script RT reagent kit (TaKaRa, Dalian, China) with a 20 µL system.

### RT-qPCR analysis

Eight candidate celery genes, *ACTIN*, *eIF*-4*α*, *GAPDH*, *TBP*, *TUB-A*, *UBC*, *TUB-B*, and *EF*-1*α*, were used to screen the appropriate reference genes under different abiotic stresses and hormone treatments based on the celery transcriptome and genome data (Li et al., 2014; Li et al., 2016b; Feng et al., 2018a). The primer sequences of *ACTIN*, *GAPDH*, *TBP*, *TUB-A*, *UBC* were consistent with previous study (Li et al., 2016b). The gene sequences of *eIF*-4*α*, *TUB-B*, and *EF*-1*α* cloned from ‘Jinnan Shiqin’ were different from the previous study, which were listed in Table S1. The RT-qPCR primer sequences of *TUB-B*, *eIF*-4*α*, and *EF*-1*α* genes were re-designed by using Primer Premier 6.0 software. The gene information and primer sequences were listed in Table 1. The specificity and accuracy of primers were determined by the PCR assay and single peak in the melting curve of RT-qPCR assay.

**Table 1:** Primer information of candidate reference genes.

Gene	RT-qPCR primers (5′→3′) forward/reverse	Amplification efficiency (E%)	Correlation coefficient (R^2^)	References
*eIF*-4*α*	GTTCCTCTCGTGTGCTCATTACCA/ TCAACCAACATCCTGTCATCATCCTT	93.8	0.999	N/A
*TUB-B*	TGGTGGCACTGGATCTGGTATGG/ ACTTTCGGAGAAGGGAAGACTGAA	98.2	0.999	N/A
*EF*-1*α*	GCTCCAGTTCTTGATTGCCACACTA/ TCATCTTAACGAATCCAGCATCACCAT	94.8	0.996	N/A
*ACTIN*	AGAAGTCCTGTTCCAGCCGTCTT/ CGAACCACCACTGAGCACTATGTT	100.7	0.998	Li et al. (2016b)
*GAPDH*	CAAGGACTGGAGAGGTGGAAGAG/ GTGAGGTCAACAACTGAGACATCC	96.8	0.998	Li et al. (2016b)
*TBP*	CTGGAGCAAAGAGCGAACAACAAT/ GCAAGACCTTCAAGCCTGATGG	109.7	0.996	Li et al. (2016b)
*TUB-A*	CCTCACCACAGGTCTCAACTTCAG/ GGTGTAGGTTGGACGCTCAATGT	92.0	0.992	Li et al. (2016b)
*UBC*	AGGCTTGAGATTCGCTGTCTGTAA/ TATTCCTGGAGCTGGCTCACTGA	101.9	0.992	Li et al. (2016b)

The 10-fold, 10^2^-fold, 10^3^-fold, 10^4^-fold, 10^5^-fold, and 10^6^-fold diluted cDNA were used to calculate the amplification efficiency (E) and correlation coefficient (R^2^), respectively (Fig. S1). The 16-fold diluted cDNA was used for RT-qPCR analysis. RT-qPCR assay was performed with the SYBR Premix *Ex Taq* (TaKaRa, Dalian, China) with a 20 µL system. The reaction volume contained 10 µL of SYBR Green I Mix, 7.2 µL of deionized water, 2 µL of diluted cDNA, and 0.4 µL of forward and reverse primers. The program of RT-qPCR assay was followed our previous study (Feng et al., 2018c).

### Data analysis

As for the primers of *eIF*-4 *α*, *TUB-B*, and *EF*-1*α* genes, the standard curves were established based on the Cq values of different dilution gradient with their corresponding logarithm of dilution multiples. The slope of standard curve was used to calculate the amplification efficiency (E) of primers, according to the formula: E% = (10^[−1∕*slope*]^-1) ×100% (Radonic et al., 2004). The amplification efficiency of other primers was reported in previous study (Li et al., 2016b).

The stabilities of candidate reference genes under different treatments were ranked based on the analysis results of geNorm (Vandesompele et al., 2002), NormFinder (Andersen, Jensen & Orntoft , 2004), BestKeeper (Pfaffl et al., 2004), ΔCt (Silver et al., 2006), and RefFinder (Xie et al., 2012). Nine Cq values were obtained from each sample, including three biological and three technical replicates. Before geNorm and NormFinder analysis, the raw Cq values should be calculated with the 2^−ΔCt^ formula (ΔCt indicated the Cq value of the sample minus the minimum Cq value). The geNorm program calculated the M value of each gene, and two genes with the lowest M value were the most stable reference genes. The pairwise variation (Vn/n+1) in geNorm program indicated the optimal number of reference gene for the normalization of RT-qPCR. If the Vn/n+1 value <0.15, the optimal number of optimal internal reference genes is n; adversely, the optimal number of reference genes is *n* + 1. NormFinder ranked the candidate genes according to the stability value calculated by the expression variations of intragroup and the intergroup of each gene. In BestKeeper, the standard deviation (SD) and coefficient of variation (CV) were calculated based on the Cq values. BestKeeper ranked the stability of candidate genes according to the SD and CV values. The lowest SD and CV values indicated the most stable reference gene. The expression stabilities of ‘pairs of genes’ were compared by ΔCt program. Based on the analysis results of various programs, the stability of candidate reference genes were comprehensively evaluated and ranked by using RefFinder.

### Validation of reference genes

Catalase was involved in plant regulatory mechanism under abiotic stress (Hu et al., 2010). Based on the protein sequence of AtCAT2 (GenBank accession number NP_195235.1) and our transcriptome and genome data, the celery *CAT1* gene was identified and cloned from ‘Jinnan Shiqin’. The sequence of *CAT1* has been submitted to GenBank (accession number:  MN365877). The RT-qPCR primer of *CAT1* was designed by using Premier 6.0 software according to the gene sequence (forward: 5′-TTCACCTTCCTCTTGGATGACATTGG-3′and reverse: 5′-GCTCCTCCGATCTTGATGGCTTC-3′). The RT-qPCR assay of *CAT1* gene under drought treatment was conducted to validate the candidate reference genes. The relative expression level of *CAT1* gene was normalized using various reference genes according to the 2^−ΔΔ*Ct*^ method (Schmittgen & Livak, 2008).

## Results

### Selection of candidate reference genes

Eight genes, *ACTIN*, *eIF*-4*α*, *GAPDH*, *TBP*, *TUB-A*, *UBC*, *TUB-B*, and *EF*-1*α* were selected as candidate reference genes from celery. The specificity and efficiency of primers were confirmed by PCR amplification assay and melting curve of RT-qPCR. The single band corresponding to various reference genes was detected in the electrophoretogram with 1.5% agarose gel, respectively (Fig. S2). The melting curves showed that candidate reference genes had a single peak in RT-qPCR reaction (Fig. S3). The amplification efficiency (E) and correlation coefficient (R^2^) of the candidate genes meets the standard of RT-qPCR assay (90% <E % <110%; R^2^ > 0.99; Table 1) (Li et al., 2016b).

### RT-qPCR results of candidate reference genes

**Figure 1:**
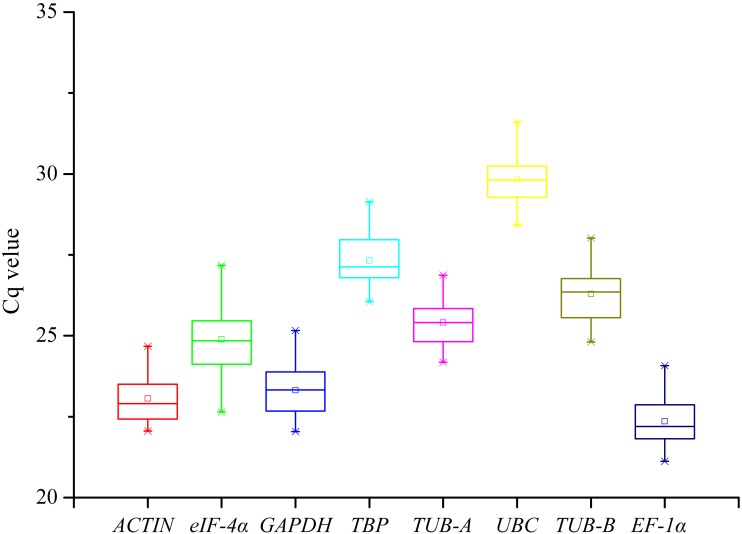
The distribution of Cq values of eight candidate reference genes in all samples from the RT-qPCR assay.

**Figure 2:**
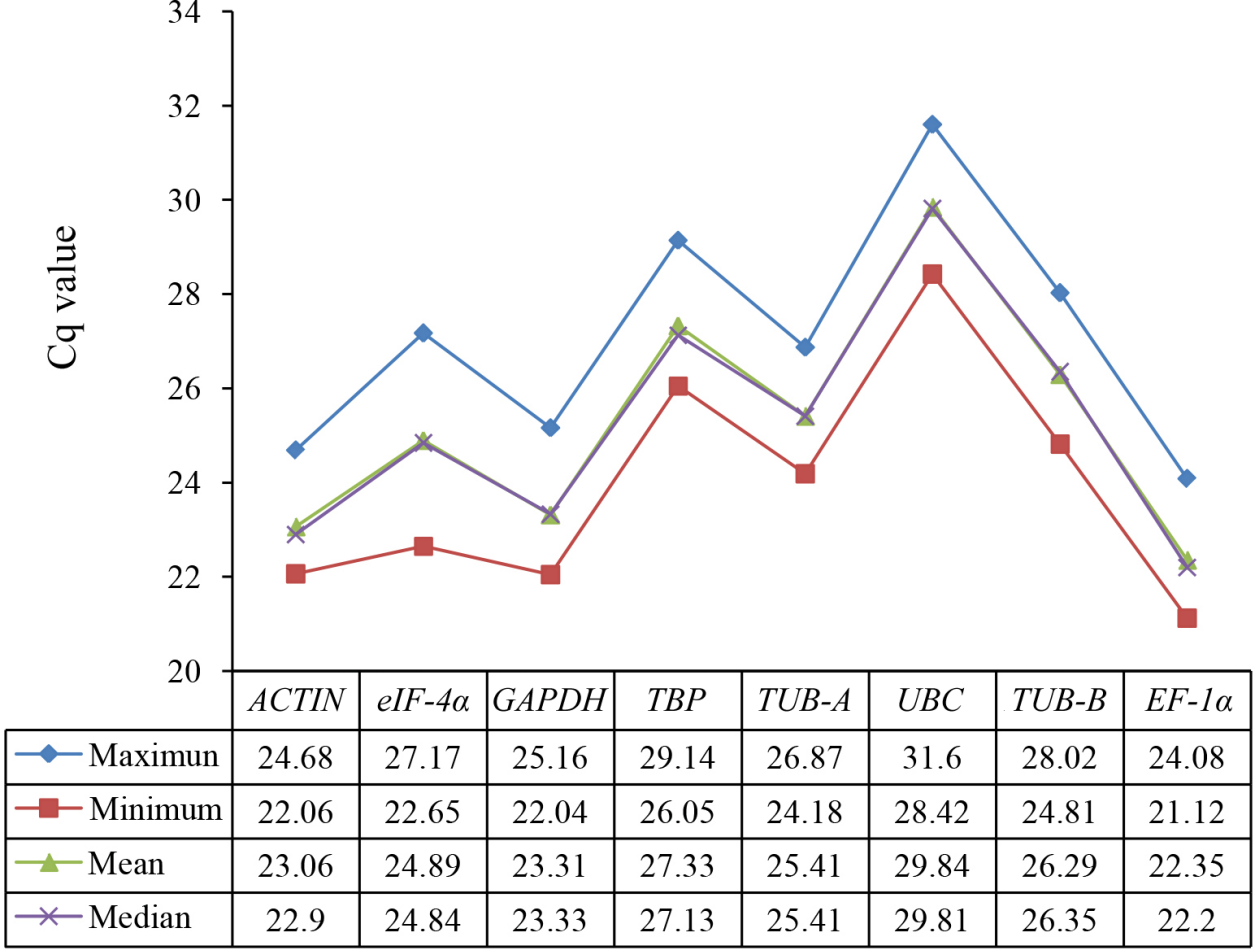
Statistic analysis (maximum, minimum, mean, and median) of Cq values of eight candidate reference genes in all samples.

The gene expression levels were indicated with the Cq values in RT-qPCR assay. The raw Cq values of candidate reference genes were listed in Table S2 and their statistics were demonstrated in Fig. 1. The Cq values of 8 candidate reference genes under different treatments ranged from 21.12 (*EF*-1*α* under SA) to 31.60 (*UBC* under GA). Low Cq values represent high expression levels, whereas high Cq values represent low expression levels. The *EF*-1*α* showed the highest expression level with the lowest average Cq value (22.35), followed by *ACTIN* (23.06), *GAPDH* (23.31), *eIF*-4*α* (24.89), *TUB-A* (25.41), *TUB-B* (26.29), *TBP* (27.33), and *UBC* (29.84) (Fig. 2).

### Stability analysis of candidate reference genes

Five programs, geNorm, NormFinder, BestKeeper, ΔCt, and RefFinder were used to determine the expression stability of the celery candidate reference genes. To evaluate the gene stabilities under different treatments, celery plants were subjected with 8 treatments, abiotic stresses (heat, cold, drought, and salt) and hormone treatments (SA, MeJA, GA, and ABA). In addition to the stability analysis of single-treatment, these 8 treatments were also divided into three groups, namely abiotic stress (heat, cold, drought, and salt), hormone stimuli (GA, SA, ABA, and MeJA), and total (all treatments), for stability analysis.

#### geNorm analysis

geNorm program calculated the M values of candidate reference genes and ranked their stabilities based on the M values. The lowest M value represents the most stable expression stability. As shown in Table 2, the M value of reference genes under all treatments were less than the default limit (1.5), which indicated that their expression stability were satisfactory. *EF*-1*α* and *TBP* genes were the most stable reference genes with the lowest M value under cold, drought, SA, and GA treatments. Meanwhile, *TBP* was also showed the highest expression stability under heat and MeJA treatments. Under salt treatment, *ACTIN* and *TUB-A* showed the lowest M value and they were the most stable reference genes. As for the abiotic stress and total groups, the M values of *ACTIN* and *EF*-1*α* were the lowest, which indicated that they were the most stable reference genes (Table 3). As for the hormone stimuli group, *TBP* and *GAPDH* showed higher high expression stability than others. In the stability analysis of all three groups, *TUB-B* was the worst stable reference gene with the lowest M value.

**Table 2:** The expression stability of candidate reference genes under single treatments calculated by geNorm, NormFinder, BestKeeper, Δ*Ct*, and RefFinder.

Treatments	Rank	geNorm		NormFinder		BestKeeper			ΔCt		RefFinder
		Gene	Stability	Gene	Stability	Gene	SD	CV	Gene	Stability	Gene
Heat	1	*TBP*	0.27	*ACTIN*	0.12	*GAPDH*	0.67	2.84	*ACTIN*	0.50	*ACTIN*
	2	*UBC*	0.27	*TUB-A*	0.13	*TUB-A*	0.72	2.77	*TUB-A*	0.52	*GAPDH*
	3	*GAPDH*	0.33	*GAPDH*	0.22	*UBC*	0.76	2.57	*GAPDH*	0.55	*TUB-A*
	4	*TUB-A*	0.44	*EF*-1*α*	0.31	*TBP*	0.76	2.78	*EF*-1*α*	0.62	*TBP*
	5	*ACTIN*	0.47	*TBP*	0.34	*ACTIN*	0.80	3.41	*TBP*	0.63	*UBC*
	6	*EF*-1*α*	0.52	*eIF*-4*α*	0.37	*EF*-1*α*	0.82	3.61	*UBC*	0.65	*EF*-1*α*
	7	*eIF*-4*α*	0.55	*UBC*	0.37	*eIF*-4*α*	0.99	3.95	*eIF*-4*α*	0.70	*eIF*-4*α*
	8	*TUB-B*	0.63	*TUB-B*	0.54	*TUB-B*	1.05	4.00	*TUB-B*	0.85	*TUB-B*
Cold	1	*EF*-1*α*	0.20	*EF*-1*α*	0.04	*TUB-B*	0.47	1.77	*TBP*	0.35	*EF*-1*α*
	2	*TBP*	0.20	*ACTIN*	0.05	*TUB-A*	0.49	1.89	*EF*-1*α*	0.35	*TBP*
	3	*ACTIN*	0.21	*TBP*	0.08	*EF*-1*α*	0.49	2.19	*ACTIN*	0.36	*ACTIN*
	4	*GAPDH*	0.24	*TUB-A*	0.16	*GAPDH*	0.51	2.17	*GAPDH*	0.42	*TUB-A*
	5	*UBC*	0.27	*UBC*	0.20	*UBC*	0.55	1.83	*TUB-A*	0.42	*GAPDH*
	6	*TUB-A*	0.30	*GAPDH*	0.20	*ACTIN*	0.58	2.50	*UBC*	0.43	*TUB-B*
	7	*eIF*-4*α*	0.37	*eIF*-4*α*	0.38	*TBP*	0.60	2.15	*eIF*-4*α*	0.61	*UBC*
	8	*TUB-B*	0.46	*TUB-B*	0.49	*eIF*-4*α*	0.84	3.34	*TUB-B*	0.74	*eIF*-4*α*
Drought	1	*EF*-1*α*	0.18	*EF*-1*α*	0.03	*UBC*	0.22	0.73	*EF*-1*α*	0.38	*EF*-1*α*
	2	*TBP*	0.18	*TBP*	0.09	*TUB-A*	0.33	1.27	*TBP*	0.38	*TBP*
	3	*ACTIN*	0.22	*ACTIN*	0.11	*TBP*	0.40	1.43	*ACTIN*	0.41	*ACTIN*
	4	*GAPDH*	0.25	*TUB-A*	0.21	*EF*-1*α*	0.43	1.86	*GAPDH*	0.47	*TUB-A*
	5	*TUB-A*	0.31	*GAPDH*	0.23	*ACTIN*	0.44	1.84	*TUB-A*	0.48	*UBC*
	6	*UBC*	0.35	*UBC*	0.28	*GAPDH*	0.47	1.94	*UBC*	0.52	*GAPDH*
	7	*eIF*-4*α*	0.43	*eIF*-4*α*	0.38	*TUB-B*	0.55	2.03	*eIF*-4*α*	0.64	*eIF*-4*α*
	8	*TUB-B*	0.50	*TUB-B*	0.45	*eIF*-4*α*	0.63	2.44	*TUB-B*	0.71	*TUB-B*
Salt	1	*ACTIN*	0.22	*ACTIN*	0.08	*EF*-1*α*	0.54	2.40	*ACTIN*	0.43	*ACTIN*
	2	*TUB-A*	0.22	*TBP*	0.13	*TUB-B*	0.59	2.22	*TBP*	0.45	*TUB-A*
	3	*EF*-1*α*	0.28	*TUB-A*	0.14	*TUB-A*	0.59	2.30	*TUB-A*	0.48	*EF*-1*α*
	4	*TBP*	0.32	*EF*-1*α*	0.17	*UBC*	0.60	2.00	*EF*-1*α*	0.48	*TBP*
	5	*GAPDH*	0.36	*UBC*	0.21	*ACTIN*	0.66	2.84	*UBC*	0.51	*UBC*
	6	*UBC*	0.37	*GAPDH*	0.25	*TBP*	0.81	2.95	*GAPDH*	0.52	*TUB-B*
	7	*eIF*-4*α*	0.46	*eIF*-4*α*	0.49	*GAPDH*	0.83	3.56	*eIF*-4*α*	0.77	*GAPDH*
	8	*TUB-B*	0.56	*TUB-B*	0.56	*eIF*-4*α*	1.09	4.37	*TUB-B*	0.86	*eIF*-4*α*
SA	1	*EF*-1*α*	0.17	*EF*-1*α*	0.06	*UBC*	0.60	2.00	*ACTIN*	0.34	*TBP*
	2	*TBP*	0.17	*ACTIN*	0.06	*TUB-B*	0.70	2.67	*TBP*	0.35	*EF*-1*α*
	3	*ACTIN*	0.18	*TBP*	0.07	*TBP*	0.74	2.69	*EF*-1*α*	0.35	*ACTIN*
	4	*GAPDH*	0.24	*TUB-A*	0.17	*ACTIN*	0.77	3.33	*TUB-A*	0.42	*UBC*
	5	*TUB-A*	0.28	*GAPDH*	0.22	*TUB-A*	0.79	3.07	*GAPDH*	0.44	*TUB-A*
	6	*UBC*	0.32	*UBC*	0.26	*EF*-1*α*	0.84	3.78	*UBC*	0.49	*GAPDH*
	7	*eIF*-4*α*	0.38	*eIF*-4*α*	0.36	*GAPDH*	0.86	3.68	*eIF*-4*α*	0.59	*TUB-B*
	8	*TUB-B*	0.46	*TUB-B*	0.44	*eIF*-4*α*	1.04	4.16	*TUB-B*	0.68	*eIF*-4*α*
MEJA	1	*TBP*	0.28	*EF*-1*α*	0.09	*ACTIN*	0.69	3.00	*ACTIN*	0.46	*EF*-1*α*
	2	*GAPDH*	0.28	*ACTIN*	0.09	*eIF*-4*α*	0.86	3.42	*EF*-1*α*	0.49	*ACTIN*
	3	*ACTIN*	0.32	*TBP*	0.21	*GAPDH*	0.76	3.27	*TBP*	0.52	*TBP*
	4	*EF*-1*α*	0.34	*GAPDH*	0.22	*TBP*	0.82	2.99	*GAPDH*	0.53	*GAPDH*
	5	*TUB-A*	0.40	*TUB-A*	0.35	*TUB-A*	0.96	3.79	*TUB-A*	0.65	*UBC*
	6	*eIF*-4*α*	0.47	*eIF*-4*α*	0.36	*UBC*	0.33	1.11	*eIF*-4*α*	0.68	*TUB-A*
	7	*UBC*	0.53	*UBC*	0.42	*TUB-B*	0.85	3.24	*UBC*	0.73	*eIF*-4*α*
	8	*TUB-B*	0.61	*TUB-B*	0.54	*EF*-1*α*	0.62	2.74	*TUB-B*	0.86	*TUB-B*
GA	1	*EF*-1*α*	0.17	*EF*-1*α*	0.05	*TBP*	0.35	1.24	*EF*-1*α*	0.39	*EF*-1*α*
	2	*TBP*	0.17	*ACTIN*	0.08	*EF*-1*α*	0.35	1.50	*ACTIN*	0.40	*TBP*
	3	*GAPDH*	0.23	*TBP*	0.11	*ACTIN*	0.38	1.58	*TBP*	0.41	*ACTIN*
	4	*ACTIN*	0.27	*GAPDH*	0.25	*GAPDH*	0.42	1.72	*GAPDH*	0.50	*GAPDH*
	5	*TUB-A*	0.37	*eIF*-4*α*	0.32	*TUB-A*	0.42	1.62	*TUB-A*	0.59	*TUB-A*
	6	*eIF*-4*α*	0.43	*TUB-A*	0.33	*TUB-B*	0.50	1.89	*eIF*-4*α*	0.59	*eIF*-4*α*
	7	*TUB-B*	0.47	*TUB-B*	0.35	*UBC*	0.54	1.76	*TUB-B*	0.61	*TUB-B*
	8	*UBC*	0.52	*UBC*	0.39	*eIF*-4*α*	0.57	2.22	*UBC*	0.65	*UBC*
ABA	1	*EF*-1*α*	0.23	*EF*-1*α*	0.05	*UBC*	0.33	1.09	*EF*-1*α*	0.38	*EF*-1*α*
	2	*GAPDH*	0.23	*TBP*	0.15	*GAPDH*	0.53	2.24	*TBP*	0.42	*GAPDH*
	3	*TBP*	0.27	*GAPDH*	0.20	*EF*-1*α*	0.54	2.40	*GAPDH*	0.45	*TBP*
	4	*ACTIN*	0.34	*TUB-A*	0.20	*eIF*-4*α*	0.59	2.30	*TUB-A*	0.46	*UBC*
	5	*TUB-A*	0.36	*ACTIN*	0.21	*TBP*	0.62	2.22	*ACTIN*	0.46	*TUB-A*
	6	*UBC*	0.42	*eIF*-4*α*	0.34	*TUB-A*	0.62	2.42	*UBC*	0.59	*ACTIN*
	7	*eIF*-4*α*	0.46	*UBC*	0.34	*ACTIN*	0.71	3.06	*eIF*-4*α*	0.60	*eIF*-4*α*
	8	*TUB-B*	0.49	*TUB-B*	0.36	*TUB-B*	0.77	2.94	*TUB-B*	0.60	*TUB-B*

**Table 3:** The expression stability of candidate reference genes under three groups calculated by geNorm, NormFinder, BestKeeper, ΔCt, and RefFinder.

Group	Rank	geNorm		NormFinder		BestKeeper				ΔCt		RefFinder
		Gene	Stability	Gene	Stability	Gene	SD		CV		Gene	Stability	Gene
Abiotic stress	1	*ACTIN*	0.24	*ACTIN*	0.13	*TUB-A*	0.49		1.92		*ACTIN*	0.44	*ACTIN*
	2	*EF*-1*α*	0.24	*TUB-A*	0.14	*UBC*	0.55		1.87		*TUB-A*	0.45	*TUB-A*
	3	*TUB-A*	0.30	*GAPDH*	0.19	*EF*-1*α*	0.59		2.60		*GAPDH*	0.47	*EF*-1*α*
	4	*GAPDH*	0.35	*TBP*	0.21	*GAPDH*	0.60		2.56		*TBP*	0.48	*GAPDH*
	5	*TBP*	0.37	*EF*-1*α*	0.22	*TBP*	0.60		2.20		*EF*-1*α*	0.49	*TBP*
	6	*UBC*	0.41	*UBC*	0.30	*TUB-B*	0.65		2.44		*UBC*	0.56	*UBC*
	7	*eIF*-4*α*	0.48	*eIF*-4*α*	0.41	*ACTIN*	0.65		2.80		*eIF*-4*α*	0.68	*eIF*-4*α*
	8	*TUB-B*	0.53	*TUB-B*	0.42	*eIF*-4*α*	0.92		3.68		*TUB-B*	0.69	*TUB-B*
Hormone stimuli	1	*TBP*	0.30	*EF*-1*α*	0.10	*UBC*	0.54		1.78		*EF*-1*α*	0.43	*EF*-1*α*
	2	*GAPDH*	0.30	*ACTIN*	0.13	*TUB-A*	0.65		2.55		*ACTIN*	0.44	*ACTIN*
	3	*EF*-1*α*	0.35	*TBP*	0.22	*EF*-1*α*	0.65		2.92		*GAPDH*	0.48	*TBP*
	4	*ACTIN*	0.36	*GAPDH*	0.22	*ACTIN*	0.66		2.85		*TBP*	0.49	*GAPDH*
	5	*TUB-A*	0.39	*TUB-A*	0.28	*TUB-B*	0.66		2.54		*TUB-A*	0.54	*TUB-A*
	6	*eIF*-4*α*	0.45	*eIF*-4*α*	0.33	*eIF*-4*α*	0.72		2.86		*eIF*-4*α*	0.60	*UBC*
	7	*UBC*	0.48	*UBC*	0.33	*TBP*	0.75		2.74		*UBC*	0.60	*eIF*-4*α*
	8	*TUB-B*	0.53	*TUB-B*	0.40	*GAPDH*	0.77		3.29		*TUB-B*	0.67	*TUB-B*
Total	1	*ACTIN*	0.28	*ACTIN*	0.16	*TUB-A*	0.55		2.15		*ACTIN*	0.47	*ACTIN*
	2	*EF*-1*α*	0.28	*EF*-1*α*	0.18	*UBC*	0.58		1.96		*EF*-1*α*	0.49	*EF*-1*α*
	3	*TUB-A*	0.34	*GAPDH*	0.19	*EF*-1*α*	0.59		2.63		*GAPDH*	0.50	*TUB-A*
	4	*GAPDH*	0.39	*TBP*	0.23	*ACTIN*	0.62		2.68		*TBP*	0.52	*GAPDH*
	5	*TBP*	0.41	*TUB-A*	0.25	*TBP*	0.65		2.37		*TUB-A*	0.54	*TBP*
	6	*eIF*-4*α*	0.48	*eIF*-4*α*	0.37	*GAPDH*	0.68		2.90		*eIF*-4*α*	0.66	*UBC*
	7	*UBC*	0.53	*UBC*	0.40	*TUB-B*	0.70		2.65		*UBC*	0.68	*eIF*-4*α*
	8	*TUB-B*	0.57	*TUB-B*	0.41	*eIF*-4*α*	0.80		3.20		*TUB-B*	0.70	*TUB-B*

**Figure 3:**
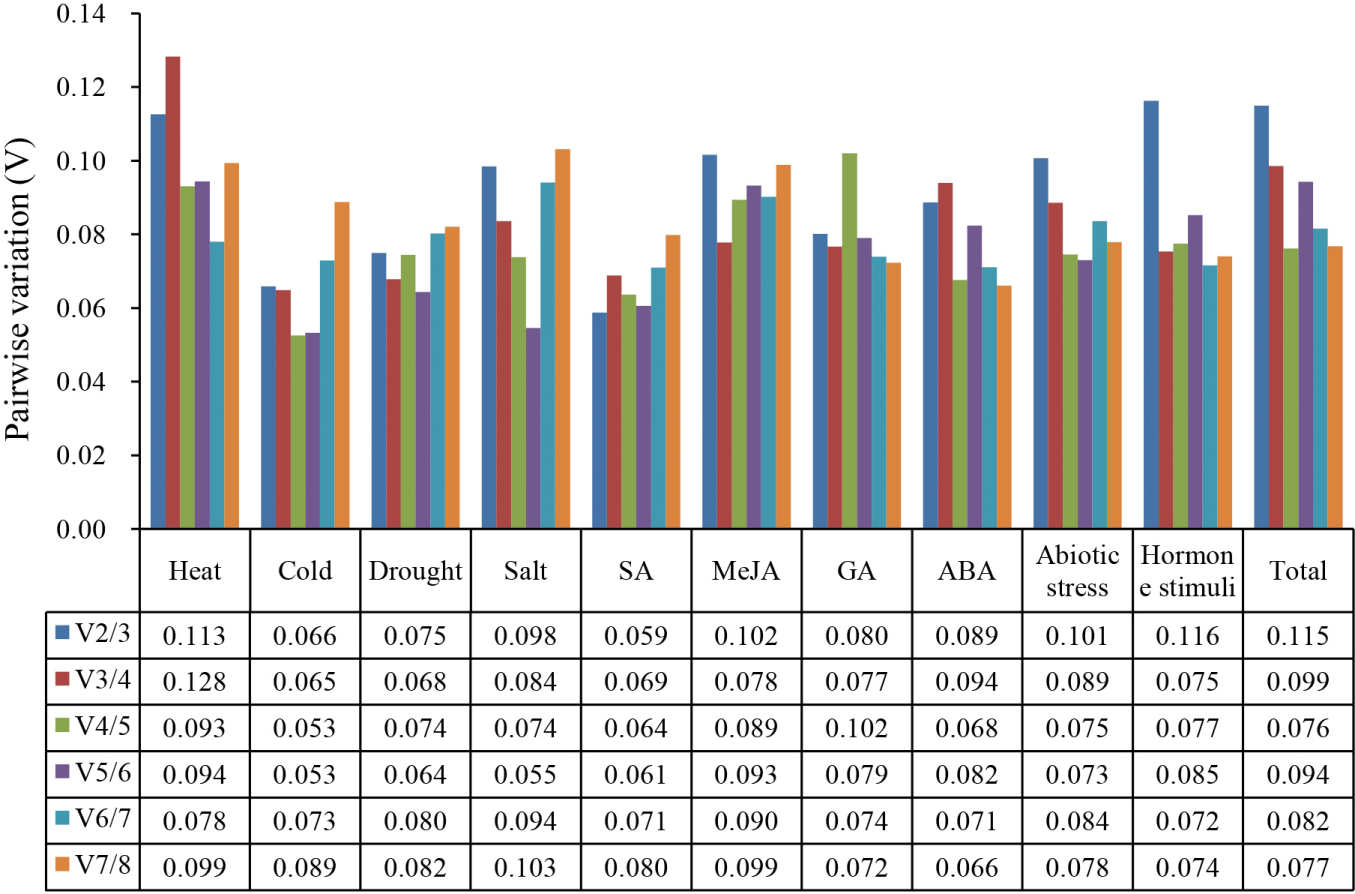
Calculation of optimal number of reference gene during gene normalization by pairwise variation from geNorm.

In RT-qPCR analysis, multiple reference genes can be selected to quantify the expression of the target gene with more accuracy. The pairwise variations (Vn/n+1) calculated by geNorm program were used to determine the optimal number of reference genes in the normalization. As shown in Fig. 3, the V2/3 values of eight single-treatment and the three treatment groups were less than 0.15. Therefore, two suitable reference genes were adequate for gene expression normalization under the above treatments.

#### NormFinder analysis

NormFinder program ranked the reference genes based on the calculated stability values in different experimental designs. As shown in Tables 2 and 3, *ACTIN* was the most stable reference gene under heat and salt treatments, and *EF*-1*α* was the most stable gene under cold, drought and all tested hormone treatments. When performing stability analysis among multiple treatments, *ACTIN* was the most stable reference gene with the lowest stability value in both the abiotic stress group and the total group. As for the hormone stimuli group, the expression stability of *EF*-1*α* gene was the highest, followed by *ACTIN* gene. Similar to the geNorm analysis, *eIF*-4*α*, *UBC,* and *TUB-B* were the worst stable reference genes in the NormFinder analysis.

#### BestKeeper analysis

The BestKeeper program ranked the reference genes based on the SD and CV of Cq values in the RT-qPCR assay. Low SD and CV values represent the high expression stability. *UBC* was the most stable reference gene under drought, SA, and ABA treatments. *EF*-1*α* and *ACTIN* were the most stable reference genes under salt and MeJA treatments, respectively. As for the abiotic stress group and the total group, *TUB-A* showed the highest expression stability, and *eIF*-4*α* showed the lowest expression stability. *UBC* was the most stable reference gene and *GAPDH* was the least stable reference gene in hormone stimuli group.

#### ΔCt analysis

The expression stability of the eight candidate gene was calculated and ranked based on the ΔCt method. In single treatment, *ACTIN* was the most stable expressed reference gene under heat, salt, SA, and MEJA treatments, respectively. As for drought, GA, and ABA treatments, *EF*-1*α* gene was the best reference gene for gene normalization. The expression stability under different groups was also investigated. *ACTIN* gene showed the most expression stability under abiotic stress treatments. In hormone stimuli and total groups, the expressions of *ACTIN* and *EF*-1*α* genes showed the highest stabilities. In addition, ΔCt analysis results indicated that *TUB-B* was the worst reference gene under most treatments.

#### RefFinder analysis

Considering the results of all statistic methods, the stability of those celery candidate reference genes was comprehensively evaluated by RefFinder. As shown in Table 2, *ACTIN* gene was the most stable reference gene under heat and salt treatments. *EF*-1*α* showed the most stable expression under cold, drought, MEJA, GA, and ABA treatments. As for the stability analysis in different groups, *ACTIN* gene was the most stable reference gene and *TUB-B* was the worst stable reference gene in the abiotic stress, hormone stimuli, and total groups (Table 3).

### Validation of reference genes

**Figure 4:**
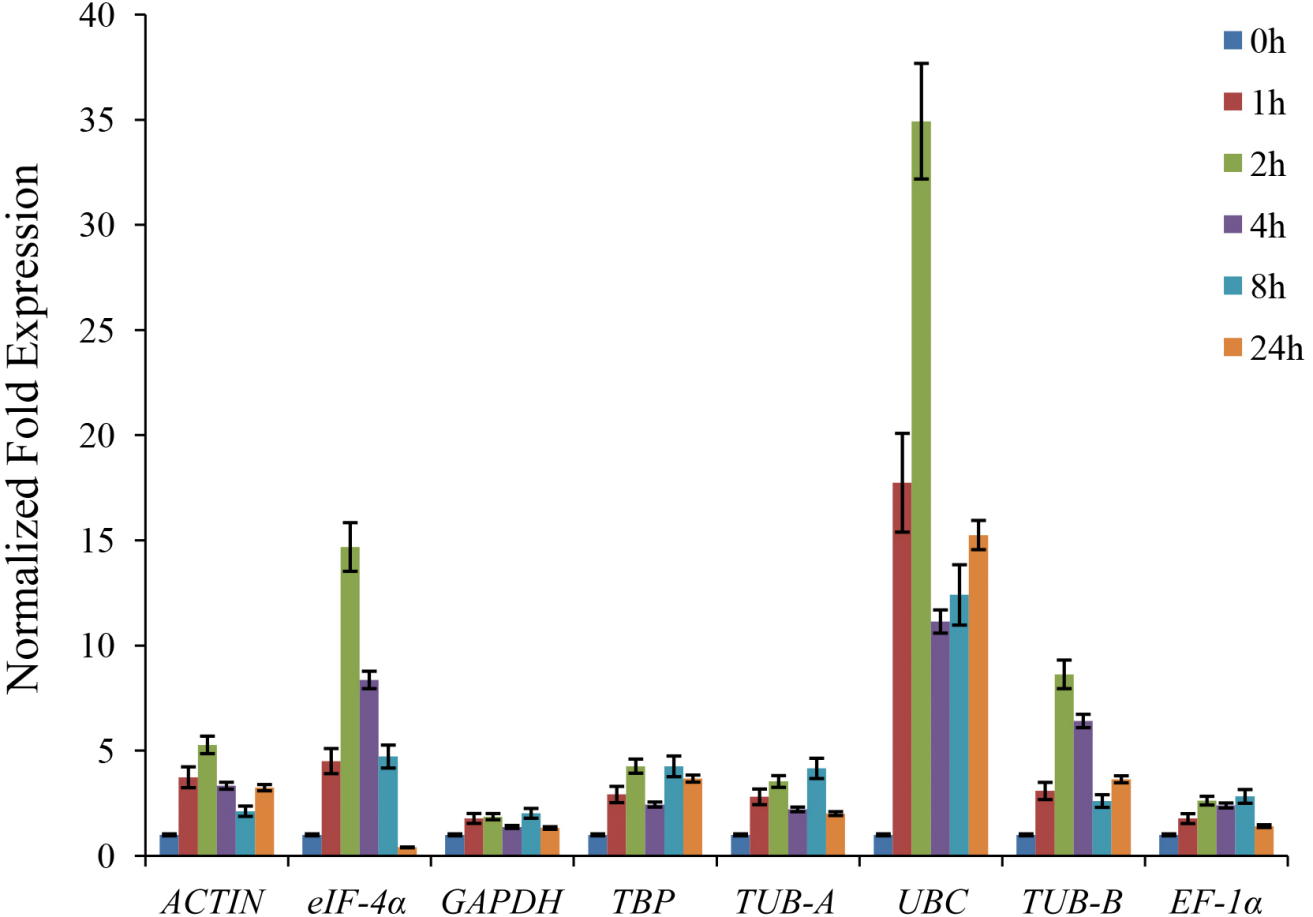
The relative expression levels of *CAT* gene normalized by different reference genes under drought stress.

The *CAT* gene encoded the catalase, which is involved in the regulation of stress defense in plants (Willekens et al., 1997). The expression of the *CAT* gene could be induced by many abiotic stresses, including chilling, drought, and salt (Fadzillah et al., 1996; Kim et al., 2007). The celery *CAT1* gene was cloned from the cDNA of ‘Jinnan Shiqin’ and sequenced. In this study, the relative expression level of *CAT1* gene under drought stress was detected to validate the reference genes. As shown in Fig. 4, the expression levels of *CAT1* gene normalized by various reference genes were different. The expression levels of *CAT1* gene under drought stress were increased using *ACTIN*, *GAPDH*, *TBP*, *TUB-A*, *UBC*, *TUB-B*, and *EF*-1*α* as reference gene, respectively. When used the unstable reference gene *eIF*-4*α* for normalization, the expression of the *CAT1* gene was decreased after 24 h of drought treatment.

## Discussion

Gene expression plays important roles in plant development and environmental stimuli defense. Research on gene expression contributed to unravel the complex regulatory mechanisms in life cycle of plant (Feng et al., 2018b; Silva et al., 2019). Nowadays, RT-qPCR is a general technique to determine the expression level of target gene (Gachon, Mingam & Charrier , 2004). However, the accuracy of RT-qPCR results was affected by many factors. Using proper reference gene was an effective approach to improve the accuracy of gene normalization during RT-qPCR assay (Nicot et al., 2005; Gutierrez et al., 2008). A previous study has investigated the suitable reference genes among various tissues and development stages in celery (Li et al., 2016b). In the process of growth and development, celery also encounters many environmental stimuli, including biotic and abiotic stresses. Selection of suitable reference genes is crucial to normalize the gene expression under different conditions in celery. The current study evaluated the expression stabilities of various candidate reference genes under abiotic stress and hormone stimuli in celery.

In this work, the expression profiles of 8 candidate reference genes of celery (*ACTIN*, *eIF*-4 *α*, *GAPDH*, *TBP*, *TUB-A*, *UBC*, *TUB-B*, and *EF*-1 *α*) under different abiotic stress and hormone stimuli were determined and compared. These candidate genes were commonly used for the selection of appropriate reference genes under different treatments in other species (Tian et al., 2015; Li et al., 2016a; Lu et al., 2018). The specificity and amplification efficiency of primers were confirmed and met the basic requirements of RT-qPCR (Ramakers et al., 2003). In the RT-qPCR assay, the ranges of Cq values among various candidate reference gene were different. Based on the raw Cq values, *EF*-1*α* gene showed the highest transcript abundance and *UBC* gene showed the lowest transcript abundance.

In order to find the appropriate reference gene under different conditions, the expression stabilities of candidate genes were mainly evaluated and ranked by five methods, geNorm (Vandesompele et al., 2002), NormFinder (Andersen, Jensen & Orntoft , 2004), BestKeeper (Pfaffl et al., 2004), ΔCt (Silver et al., 2006), and RefFinder (Xie et al., 2012). Here, the stabilities ranking of these selected candidate reference genes of celery in five programs were not completely consistent, especially between BestKeeper and other programs. For example, the most stable reference genes under heat treatment recommended by geNorm, NormFinder, and BestKeeper were *TBP*, *ACTIN*, and *GAPDH*, respectively. In the cold treatment, *TUB-B* was the most stable reference gene under BestKeeper analysis but showed the worst stability under geNorm and NormFinder analyses. The ranking differences of various programs were mainly due to the variations in their algorithms (Ransbotyn & Reusch, 2006).

Based on the geNorm analysis, the pairwise variation values of V2/3 under all treatments were below the threshold (0.15), which indicated that two reference genes of celery were sufficient for the normalization of gene expression (Vandesompele et al., 2002). Considering the difference in various analysis methods, RefFinder was used to comprehensively evaluate the expression stability of candidate reference genes (Xie et al., 2012). *ACTIN* was the most recommended reference gene, and *TUB-B* was the worst stable gene under different treatments in celery. As the most stable reference gene in celery, *ACTIN* was also investigated to be the suitable reference gene in carrot and soybean under abiotic stress treatments (Jian et al., 2008; Tian et al., 2015). The *ACTIN* was the most stable gene at different development stages in carrot (Wang et al., 2016). It should be noted that the *TUB-B* investigated to be the most stable gene among tissues and developmental stages of celery (Li et al., 2016b), whereas our study indicated that *TUB-B* was the least stable gene under different treatments. This indicated that the stability of the same house-keeping gene was various under different conditions.

Celery generated physiological regulation through the expressions of specific genes during development and environmental stress. Plant accumulated amounts of hydrogen peroxide (H_2_O_2_) under stress conditions (Willekens et al., 1997). The *CAT* gene encoded the catalase, which is involved in the regulation of H_2_O_2_ level in plants. Previous study indicated that the expression of *CAT* gene was up-regulated under drought stress (Nie et al., 2015). To validate the reliability of reference genes, the relative expression level of *CAT1* gene was normalized by using different reference genes. Except when using the unstable reference gene *eIF*-4 *α*, the expressions of *CAT1* normalized by other reference genes were increased under drought treatment.

## Conclusions

This work aims to select the appropriate reference gene under abiotic stress and hormone stimuli in celery. The stability of eight candidate reference genes under different treatments was evaluated and ranked by geNorm, NormFinder, BestKeeper, Δ*Ct*, and RefFinder programs. The analysis results indicated that *ACTIN* was the most recommended reference gene under abiotic stress and hormone treatments in celery, whereas the *TUB-B* was the worst stable gene. The reliability of celery reference gene was verified by expression normalization of *CAT1* gene under drought stress. In conclusion, the results in this study provided reference and basis for the selection of suitable reference genes under abiotic stress and hormone treatment in celery.

##  Supplemental Information

10.7717/peerj.7925/supp-1 Figure S1 Analysis of amplification efficiency (E) and correlation coefficient (*R*^2^) of the *eIF-4α*, *TUB-B* and *EF-1α* genes

10.7717/peerj.7925/supp-2 Figure S2 PCR amplification of candidate reference genes and *CAT* gene

10.7717/peerj.7925/supp-3 Figure S3 The melting curves of candidate reference genes in RT-qPCR assay

10.7717/peerj.7925/supp-4 Table S1 Nucleotide sequences of the candidate reference genes

10.7717/peerj.7925/supp-5 Table S2 Cq values of candidate reference genes in RT-qPCR assay

## Additional Information and Declarations

### DNA Deposition

The following information was supplied regarding the deposition of DNA sequences: The CAT1 sequence is available at GenBank:  MN365877.

### Data Availability

The following information was supplied regarding data availability: The raw measurements are available in the Supplemental File.
