# Supplementary material for: Selection of appropriate reference genes for RT-qPCR analysis under abiotic stress and hormone treatment in celery
Source: PeerJ. 2019 Oct 24;7:e7925. doi: 10.7717/peerj.7925 (PMC6815649; doi:10.7717/peerj.7925)
Supplement: Figure S1 [file peerj-07-7925-s001.doc]

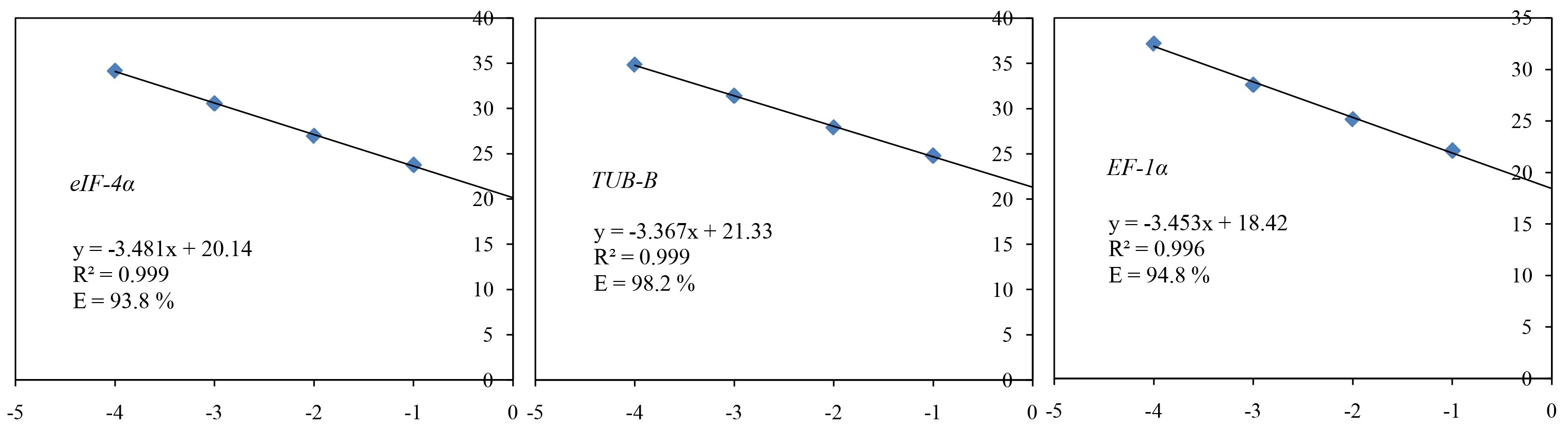


**Fig. S1** Analysis of amplification efficiency (E) and correlation coefficient (R^2^) of the *eIF-4α*, *TUB-B* and *EF-1α* genes.
