# Supplementary figures and images for: Selection of appropriate reference genes for RT-qPCR analysis under abiotic stress and hormone treatment in celery
Source: PeerJ. 2019 Oct 24;7:e7925. doi: 10.7717/peerj.7925 (PMC6815649; doi:10.7717/peerj.7925)

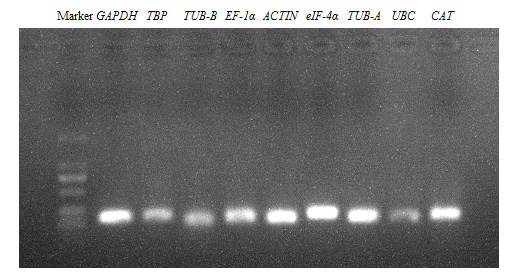


**Fig. S2** PCR amplification of candidate reference genes and *CAT* gene.

Supplement: Figure S2 [file peerj-07-7925-s002.doc]

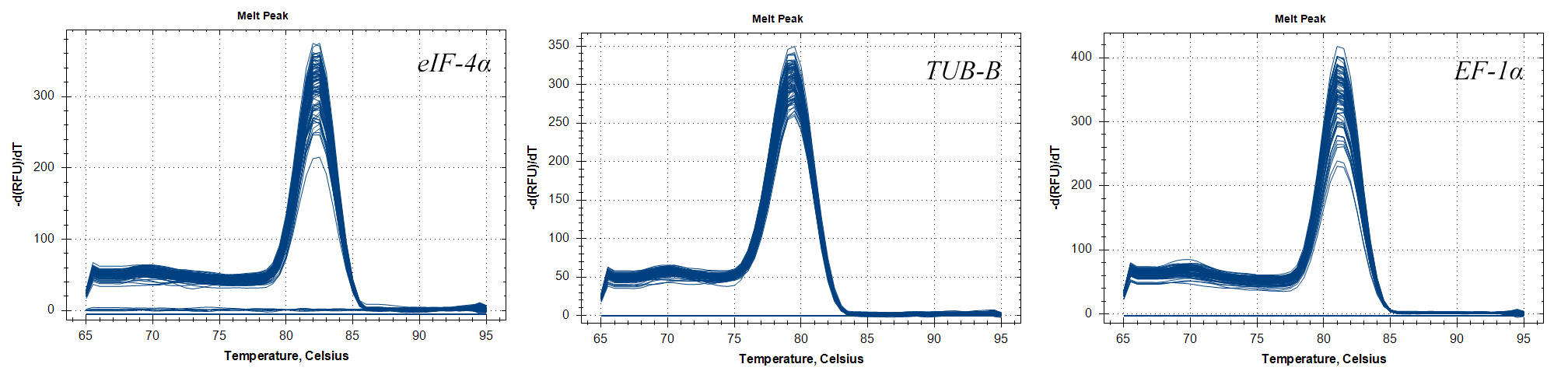


**Fig. S3** The melting curves of candidate reference genes in RT-qPCR assay.

Supplement: Figure S3 [file peerj-07-7925-s003.doc]
