# Supplementary material for: Selection of appropriate reference genes for RT-qPCR analysis under abiotic stress and hormone treatment in celery
Source: PeerJ. 2019 Oct 24;7:e7925. doi: 10.7717/peerj.7925 (PMC6815649; doi:10.7717/peerj.7925)
Supplement: Table S1 [file peerj-07-7925-s004.doc]

Table S1. Nucleotide sequences of the candidate reference genes

>eIF-4α

ATGGCTGGAGCTGCACCAGAAGGTTCTCAATTCGATGCACGTCAATTTGACGCAAAAATGACCGAGCTACTTGGTGCTGATGGAGAAGAATTCTTTACATCATATGATGAGGTTTATGACAGTTTCGATGCTATGGGACTGCAGGAAAACCTTCTGCGAGGCATCTATGCTTACGGTTTTGAGAAGCCATCTGCGATTCAGCAGCGGGGAATTGTTCCTTTCTGCAAAGGGCTAGATGTTATTCAACAGGCACAATCTGGTACTGGGAAGACAGCAACTTTCTGCTCTGGAGTTCTTCAGCAGCTTGATTTTGCTGTTGTTGAATGTCAAGCGTTGGTTCTTGCTCCTACTCGTGAACTAGCACAACAGATTGAGAAGGTTATGCGAGCACTTGGTGACTATCTTGGCGTTAAGGTTCATGCTTGTGTGGGTGGAACCAGTGTCCGCGAAGATCAGCGCATTCTCTCCAGTGGAGTTCATGTTGTGGTTGGTACTCCTGGTCGCGTGTTCGACATGCTGCGAAGACAATCTCTGCGCTCAGATTACATCAAGATGTTTGTTCTGGACGAAGCAGATGAAATGCTCTCAAGAGGATTCAAGGATCAGATTTATGATATTTTCCAGTTGTTACCTCCCAAAGTTCAGGTTGGTGTTTTCTCTGCCACCATGCCTCCTGAGGCTCTTGAAATCACAAGGAAATTCATGAATAAGCCTGTTAGGATTCTTGTGAAGCGAGATGAGCTGACTCTCGAGGGTATCAAACAATTTTATGTTAATGTTGACAAGGAGGAATGGAAACTGGAAACACTCTGTGATCTTTATGAGACTTTGGCTATTACCCAGAGTGTCATCTTTGTTAATACCAGGCGCAAGGTTGATTGGCTGACTGACAAAATGCGCAGTCGTGATCACACCGTCTCTGCCACTCATGGAGACATGGATCAGAATACTAGAGATATCATTATGCGTGAATTCCGCTCTGGTTCCTCTCGTGTGCTCATTACCACTGATCTTCTCGCTCGTGGTATAGATGTCCAGCAAGTATCACTTGTGATTAACTATGATTTGCCGACTCAGCCAGAGAATTACCTTCATCGGATTGGTCGTAGTGGACGTTTTGGAAGGAAAGGTGTTGCGATCAACTTTGTTACCAAGGATGATGACAGGATGTTGGTTGACATACAGAAGTTTTATAATGTAGTGGTCGAGGAATTGCCAGCTAATGTTGCCGATCTTCTTTAG

>TUB-B

ATGAGAGAAATCCTCCACATACAAGCAGGCCAATGTGGCAACCAAATAGGTGCCAAGTTCTGGGAAGTCATATGTGATGAACATGCAATCAACCAGTCAGGACTTTACGATGGAACAGATGATGGTCTTCAGCTGGAACGCATAAATGTTTATTATAATGAAGCTAGTGGTGGCAGATATGTTCCAAGAGCTGTGCTTGTGGACTTGGAACCTGGAACTATGGATGCTGTCAGGACAGGGCCTTATGGACAGATTTTTAGGCCTGATAATTTCGTTTTCGGACAGTCGGGTGCGGGGAATAATTGGGCTAAAGGTCATTATACCGAGGGGGCTGAGTTGGTTGATTCTGTTCTTGATGTTGTTAGAAAAGAGGCTGAGAATTGTGATTGTCTTCAAGGATTCCAAGTATGTCAGTCTCTGGGTGGTGGCACTGGATCTGGTATGGGCACCCTTCTCATTTCTAAAATTAGGGAGGAGTATCCGGATCGTATGATGTTGACATTTTCAGTCTTCCCTTCTCCGAAAGTATCTGACACTGTTGTGGAGCCATACAATGCGACGCTTTCAGTTCATCAGCTTGTTGAGAATGCTGATCAGTGCATGGTCTTGGATAATGAGGCACTGTATGACATCTGTTTCCGAACCCTCAAGCTTACGACTCCCACATTTGGTGATCTCAATCACTTGATCTCTGCTACCATGAGTGGTGTCACGTGTTGCCTTAGGTTTCCTGGTCAGCTGAACTCTGACCTACGGAAACTAGCAGTTAACCTCATTCCATTTCCGCGACTCCACTTCTTCATGGTTGGTTTTGCGCCCTTGACATCAAGAGGTTCCCAGCAATATCGTGCTCTCACTGTCCCGGAACTGACCCAGCAGATGTGGGATTCGAAGAACATGATGTGTGCTGCTGACCCAAGACATGGTCGCTACTTAACAGCGTCAGCCATGTTTCGTGGTAAGATGAGCACAAAAGAGGTTGATGAGCAATTGATTAATGTCCAGAACAAAAACTCATCATACTTTGTTGAGTGGATACCAAACAATGTCAAGTCTAGTGTCTGTGACATCCCACCAAAGGGTTTGAAAATGTCATCAACTTTTATAGGGAACTCAACCTCTATTCAGGAGATGTTCCGTCGGGTTAGTGAGCAATTCACAGCTATGTTCAGGCGAAAGGCTTTCCTGCACTGGTACACTGGTGAAGGAATGGACGAGATGGAATTCACAGAGGCCGAGAGCAACATGAATGACCTTGTGGCTGAATACCAACAATACCAGGATGCAACTGCTGAGGAGTATTACGAGGACGAAGAGGAAGCAGAACTCGAAGCTTGA

>EF-1α

ATGGGTAAGGAAAAGATTCATATCAGTATTGTGGTCATTGGCCATGTCGACTCTGGAAAGTCTACCACCACTGGTCATCTTATCTACAAGCTTGGTGGTATTGACAAGCGTGTGATCGAAAGGTTCGAGAAGGAAGCTGCTGAGATGAACAAACGTTCATTCAAGTACGCATGGGTGCTTGACAAGCTTAAGGCTGAGCGTGAACGTGGTATCACTATTGATATTGCTCTGTGGAAGTTTGAGACCACCAAGTACTACTGCACAGTTATTGATGCTCCAGGACATCGTGATTTCATTAAGAACATGATTACTGGAACTTCTCAGGCTGATTGTGCTGTCCTCATCATTGACTCCACCACTGGAGGTTTTGAAGCTGGTATCTCCAAGGATGGGCAAACTCGGGAGCACGCTCTTCTTGCATTTACACTTGGTGTCAAGCAGATGATCTGTTGCTGCAACAAGATGGATGCCACAACCCCCAAGTACTCTAAGTCTAGATTTGAAGAAATTGTGAAGGAGGTGTCATCTTATTTGAAGAAGGTTGGATACAACCCCGACAAAATTGCATTCATTCCCATCTCTGGATTTGAGGGTGACAACATGATTGATAGGTCTACCAACCTTGACTGGTACAAGGGACCAACTCTTCTTGAAGCTCTTGACCAGATCTCTGAGCCCAAAAGACCATCAGACAAGCCTCTTCGTCTCCCACTTCAGGATGTTTACAAGATTGGAGGCATTGGAACTGTGCCAGTGGGACGTGTTGAAACTGGTGTGATCAAGCCCGGTATGGTTGTGACCTTCGGTCCTTCAGGGTTGACCACTGAAGTTAAGTCTGTTGAGATGCATCACGAGGCTCTTCAGGAAGCTCTTCCTGGTGACAATGTTGGATTCAATGTTAAGAATGTTGCGGTTAAGGATCTCAAGCGTGGATATGTTGCCTCCAACTCTAAGGATGACCCTGCCAAAGAGGCTGCCAACTTCACTGCTCAAGTTATTATCATGAACCACCCTGGTCAGATTGGAAATGGTTATGCTCCAGTTCTTGATTGCCACACTAGTCACATTGCTGTTAAGTTTGCTGAAATCCAGACCAAGATTGATCGTCGTTCTGGTAAGGAGCTCGAGAAGGAGCCTAAGTTTTTGAAGAATGGTGATGCTGGATTCGTTAAGATGATTCCAACCAAGCCCATGGTGGTGGAGACCTTTATGTCCTACCCTCCTCTTGGAAGGTTTGCTGTTAGGGACATGAGGCAGACTGTTGCTGTGGGAGTCATCAAGAGTGTGGAGAAGAAGGATCCTACTGGAGCCAAGGTCACCAAGGCTGCAATCAAGAAGAAATGA
