# Supplementary material for: Selection of appropriate reference genes for RT-qPCR analysis under abiotic stress and hormone treatment in celery
Source: PeerJ. 2019 Oct 24;7:e7925. doi: 10.7717/peerj.7925 (PMC6815649; doi:10.7717/peerj.7925)
Supplement: Table S2 [file peerj-07-7925-s005.doc]

Table S2 Cq values of candidate reference genes in RT-qPCR assay.

|  | Replicates | *ACTIN* | *eIF-4α* | *GAPDH* | *TBP* | *TUB-A* | *UBC* | *TUB-B* | *EF-1α* |
| --- | --- | --- | --- | --- | --- | --- | --- | --- | --- |
| Heat-1 | 1 | 24.42 | 25.53 | 23.88 | 27.16 | 26.37 | 29.28 | 28.01 | 24.07 |
|  | 2 | 24.4 | 25.12 | 23.84 | 27.13 | 26.46 | 29.28 | 27.87 | 24.08 |
|  | 3 | 24.32 | 25.97 | 23.74 | 27.02 | 26.52 | 29.26 | 28.02 | 23.98 |
| Heat-2 | 4 | 22.13 | 23.51 | 22.36 | 26.28 | 24.63 | 28.51 | 25.19 | 21.46 |
|  | 5 | 23.03 | 23.67 | 23.34 | 27.12 | 25.32 | 29.15 | 25.28 | 21.96 |
|  | 6 | 22.06 | 23.42 | 22.24 | 26.05 | 24.44 | 28.42 | 25.14 | 21.4 |
| Heat-3 | 7 | 22.69 | 24.08 | 22.84 | 26.64 | 25.14 | 28.75 | 25.24 | 22.18 |
|  | 8 | 22.78 | 24.39 | 22.83 | 26.72 | 25.16 | 29.03 | 25.28 | 22.05 |
|  | 9 | 22.56 | 24.27 | 22.77 | 27.05 | 25.12 | 28.73 | 25.22 | 21.95 |
| Cold-1 | 1 | 22.92 | 24.55 | 23.18 | 27.17 | 25.42 | 29.32 | 26.44 | 22.17 |
|  | 2 | 23.08 | 24.35 | 23.02 | 27.22 | 25.27 | 29.18 | 26.3 | 22.01 |
|  | 3 | 22.74 | 24.53 | 23.08 | 27.13 | 25.38 | 29.04 | 26.34 | 22.07 |
| Cold-2 | 4 | 22.76 | 24.44 | 23.11 | 26.98 | 25.34 | 29.65 | 26.55 | 22.19 |
|  | 5 | 22.74 | 24.62 | 23.08 | 26.97 | 25.38 | 29.5 | 26.5 | 22.14 |
|  | 6 | 22.62 | 24.43 | 23.01 | 26.96 | 25.28 | 29.19 | 26.31 | 22.15 |
| Cold-3 | 7 | 22.77 | 24.01 | 23.38 | 27.26 | 25.58 | 29.37 | 26.47 | 22.15 |
|  | 8 | 22.78 | 24.29 | 23.41 | 27.34 | 25.63 | 29.55 | 26.62 | 22.2 |
|  | 9 | 22.96 | 24.64 | 23.56 | 27.43 | 25.81 | 29.5 | 26.67 | 22.32 |
| Drought-1 | 1 | 24.68 | 25.61 | 24.51 | 28.48 | 26.36 | 30.09 | 27.48 | 23.45 |
|  | 2 | 24.44 | 26.38 | 24.44 | 28.29 | 26.24 | 30.09 | 27.25 | 23.32 |
|  | 3 | 24.36 | 26.46 | 24.33 | 28.31 | 26.23 | 30.11 | 27.28 | 23.24 |
| Drought-2 | 4 | 23.41 | 25.24 | 23.33 | 27.53 | 25.82 | 30.3 | 26.79 | 22.43 |
|  | 5 | 23.38 | 25.44 | 23.33 | 27.63 | 25.8 | 30.17 | 27.02 | 22.53 |
|  | 6 | 23.35 | 25.31 | 23.32 | 27.61 | 25.78 | 30.29 | 26.86 | 22.41 |
| Drought-3 | 7 | 23.78 | 25.4 | 23.81 | 28.02 | 25.89 | 30.2 | 27.35 | 23.06 |
|  | 8 | 23.79 | 26.92 | 23.93 | 27.97 | 25.86 | 30.06 | 27.24 | 23.06 |
|  | 9 | 23.8 | 26.74 | 23.88 | 27.96 | 25.96 | 30.32 | 27.26 | 23.14 |
| Salt-1 | 1 | 22.84 | 24.58 | 22.89 | 27.1 | 25.59 | 30.28 | 26.76 | 22.21 |
|  | 2 | 22.78 | 24.47 | 22.73 | 26.89 | 25.41 | 29.79 | 26.71 | 22.08 |
|  | 3 | 22.68 | 24.6 | 22.7 | 27.03 | 25.59 | 29.62 | 26.54 | 22.09 |
| Salt-2 | 4 | 22.77 | 23.77 | 22.67 | 26.8 | 25.47 | 29.4 | 26.67 | 22.26 |
|  | 5 | 22.91 | 24.08 | 22.67 | 26.79 | 25.42 | 29.39 | 26.83 | 22.13 |
|  | 6 | 22.87 | 24.12 | 22.63 | 26.73 | 25.33 | 29.42 | 26.87 | 22.21 |
| Salt-3 | 7 | 22.2 | 22.65 | 22.06 | 26.17 | 24.76 | 28.87 | 25.76 | 21.83 |
|  | 8 | 22.31 | 22.95 | 22.04 | 26.09 | 24.73 | 28.58 | 25.67 | 21.8 |
|  | 9 | 22.28 | 23.24 | 22.05 | 26.13 | 24.62 | 28.67 | 25.77 | 21.78 |
| SA-1 | 1 | 22.49 | 24.11 | 22.47 | 26.96 | 24.82 | 29.25 | 26.03 | 21.57 |
|  | 2 | 22.43 | 23.86 | 22.33 | 26.86 | 24.72 | 29.13 | 26 | 21.43 |
|  | 3 | 22.36 | 24.08 | 22.28 | 26.72 | 24.73 | 29.25 | 25.91 | 21.34 |
| SA-2 | 4 | 22.19 | 23.36 | 22.29 | 26.63 | 24.74 | 29.41 | 25.43 | 21.14 |
|  | 5 | 22.11 | 23.55 | 22.3 | 26.53 | 24.69 | 29.11 | 25.37 | 21.12 |
|  | 6 | 22.12 | 24.19 | 22.29 | 26.47 | 24.7 | 29.58 | 25.57 | 21.12 |
| SA-3 | 7 | 22.52 | 24.17 | 22.67 | 26.98 | 24.9 | 29.18 | 25.25 | 21.26 |
|  | 8 | 22.55 | 24.12 | 22.67 | 27.09 | 24.97 | 29.09 | 25.27 | 21.26 |
|  | 9 | 22.49 | 24.03 | 22.66 | 26.89 | 24.98 | 29.13 | 25.25 | 21.26 |
| MeJA-1 | 1 | 22.32 | 23.94 | 22.36 | 26.36 | 24.41 | 30 | 25.73 | 21.9 |
|  | 2 | 22.33 | 23.65 | 22.24 | 26.37 | 24.22 | 29.8 | 25.59 | 21.76 |
|  | 3 | 22.14 | 23.97 | 22.14 | 26.15 | 24.18 | 29.48 | 25.56 | 21.69 |
| MeJA-2 | 4 | 22.34 | 24.42 | 22.54 | 26.88 | 24.31 | 30.29 | 25.5 | 21.78 |
|  | 5 | 22.16 | 25.02 | 22.4 | 26.57 | 24.24 | 29.91 | 25.46 | 21.62 |
|  | 6 | 22.31 | 25.03 | 22.46 | 26.69 | 24.29 | 30.24 | 25.44 | 21.62 |
| MeJA-3 | 7 | 23.15 | 25.96 | 23.37 | 26.97 | 24.96 | 30 | 27.51 | 22.71 |
|  | 8 | 23.13 | 25.33 | 23.39 | 27.01 | 24.77 | 29.97 | 27.37 | 22.7 |
|  | 9 | 23.17 | 26.01 | 23.44 | 26.96 | 24.91 | 29.74 | 27.44 | 22.68 |
| GA-1 | 1 | 23.58 | 24.99 | 24.44 | 28.25 | 25.75 | 30.02 | 26.26 | 23.03 |
|  | 2 | 23.5 | 25.04 | 24.25 | 28.01 | 25.6 | 30.08 | 26.05 | 22.69 |
|  | 3 | 23.38 | 24.96 | 24.16 | 28.39 | 25.55 | 30.11 | 25.99 | 22.66 |
| GA-2 | 4 | 23.5 | 25.55 | 24.28 | 28.41 | 25.59 | 31.12 | 26.75 | 22.91 |
|  | 5 | 23.45 | 25.55 | 24.17 | 28.31 | 25.42 | 31.6 | 26.73 | 22.87 |
|  | 6 | 23.34 | 25.46 | 24.11 | 28.31 | 25.36 | 31.39 | 26.59 | 22.69 |
| GA-3 | 7 | 23.92 | 26.21 | 24.68 | 28.9 | 25.83 | 31.52 | 26.73 | 23.13 |
|  | 8 | 23.81 | 25.99 | 24.7 | 28.71 | 25.84 | 31.25 | 26.66 | 23.14 |
|  | 9 | 23.85 | 26.22 | 24.9 | 28.56 | 25.81 | 31.1 | 26.77 | 23.13 |
| ABA-1 | 1 | 23.38 | 24.82 | 24.15 | 28.07 | 26.05 | 30.7 | 26.48 | 22.73 |
|  | 2 | 23.51 | 25.41 | 24.1 | 27.69 | 25.91 | 30.54 | 26.35 | 22.85 |
|  | 3 | 23.47 | 25.39 | 24.04 | 27.83 | 25.94 | 30.93 | 26.33 | 22.88 |
| ABA-2 | 4 | 22.12 | 25.32 | 23.33 | 27.37 | 25.25 | 30.28 | 25.75 | 22.25 |
|  | 5 | 22.2 | 25.3 | 23.48 | 27.61 | 25.35 | 30.17 | 25.69 | 22.23 |
|  | 6 | 22.8 | 25.06 | 23.43 | 27.43 | 25.23 | 30.23 | 25.67 | 22.11 |
| ABA-3 | 7 | 22.17 | 24.76 | 22.72 | 26.33 | 24.44 | 29.81 | 24.85 | 21.7 |
|  | 8 | 22.11 | 25.02 | 22.84 | 26.31 | 24.52 | 29.71 | 24.81 | 21.74 |
|  | 9 | 22.2 | 24.84 | 22.96 | 26.48 | 24.63 | 30 | 24.85 | 21.82 |
| CK-1 | 1 | 24.09 | 26.82 | 24.88 | 28.76 | 26.41 | 30.65 | 26.57 | 23.45 |
|  | 2 | 24.19 | 26.33 | 24.97 | 28.88 | 26.27 | 31.08 | 26.67 | 23.51 |
|  | 3 | 24.51 | 26.49 | 25.16 | 29.14 | 26.7 | 31.05 | 26.73 | 23.87 |
| CK-2 | 4 | 23.94 | 24.79 | 23.59 | 28.08 | 26.72 | 30.07 | 27.52 | 22.77 |
|  | 5 | 24.22 | 26.05 | 23.83 | 28.22 | 26.87 | 30.3 | 27.48 | 23.07 |
|  | 6 | 24.35 | 27.17 | 24.02 | 28.46 | 26.86 | 30.18 | 27.66 | 23.15 |
| CK-3 | 7 | 22.9 | 25.06 | 23.44 | 27.44 | 25.68 | 29.73 | 25.42 | 22.09 |
|  | 8 | 23.16 | 25.43 | 23.63 | 27.5 | 25.89 | 30.36 | 25.41 | 22.2 |
|  | 9 | 23.32 | 25.68 | 23.88 | 27.79 | 25.98 | 30.24 | 25.54 | 22.39 |
